# MicroRNAs for osteosarcoma in the mouse: a meta-analysis

**DOI:** 10.18632/oncotarget.13333

**Published:** 2016-11-12

**Authors:** Junli Chang, Min Yao, Yimian Li, Dongfeng Zhao, Shaopu Hu, Xuejun Cui, Gang Liu, Qi Shi, Yongjun Wang, Yanping Yang

**Affiliations:** ^1^ Longhua Hospital, Shanghai University of Traditional Chinese Medicine, Shanghai, China; ^2^ Spine Institute, Shanghai University of Traditional Chinese Medicine, Shanghai, China; ^3^ Division of Pulmonary and Critical Care Medicine, Department of Medicine, University of Alabama at Birmingham, Birmingham, AL, USA; ^4^ School of Rehabilitation Science, Shanghai University of Traditional Chinese Medicine, Shanghai, China

**Keywords:** miRNA, osteosarcoma, meta-analysis, mouse, therapeutic target

## Abstract

Osteosarcoma (OS) is the most common primary malignant bone carcinoma with high morbidity that happens mainly in children and young adults. As the key components of gene-regulatory networks, microRNAs (miRNAs) control many critical pathophysiological processes, including initiation and progression of cancers. The objective of this study is to summarize and evaluate the potential of miRNAs as targets for prevention and treatment of OS in mouse models, and to explore the methodological quality of current studies. We searched PubMed, Web of Science, Embase, Wan Fang Database, VIP Database, China Knowledge Resource Integrated Database, and Chinese BioMedical since their beginning date to 10 May 2016. Two reviewers separately screened the controlled studies, which estimate the effects of miRNAs on osteosarcoma in mice. A pair-wise analysis was performed. Thirty six studies with enough randomization were selected and included in the meta-analysis. We found that blocking oncogenic or restoring decreased miRNAs in cancer cells could significantly suppress the progression of OS *in vivo*, as assessed by tumor volume and tumor weight. This meta-analysis suggests that miRNAs are potential therapeutic targets for OS and correction of the altered expression of miRNAs significantly suppresses the progression of OS in mouse models, however, the overall methodological quality of studies included here was low, and more animal studies with the rigourous design must be carried out before a miRNA-based treatment could be translated from animal studies to clinical trials.

## INTRODUCTION

Osteosarcoma (OS), is a most frequent primary malignant bone tumor, accounts for 60% of all malignant childhood bone tumors, and is the second highest reason of cancer-associated death in adolescents [1, 2]. Although OS can happen in any bone, the most common sites of primary bone malignancies are the proximal tibia, proximal humerus and distal femur [1]. Typical symptoms and signs include pain history, localized swelling, joint movement limitations and typical findings of normal trabecular bone destruction on X-rays [3, 4].

Despite the neoadjuvant therapeutic strategies combined with aggressive tumor resection, the prognosis for OS patients still remains poor due to the risk of local relapse and devel­opment of pulmonary metastasis [5, 6]. For all the children diagnosed with OS, only 70% of them will survive beyond 5 years; less than 50% of them will live for more than 10 years [7-9]. Therefore, the clinical need for developing the new therapeutic approaches targeting the treatment of OS remains urgent but unmet.

MiRNAs are a class of non-coding RNAs containing about 22 nucleotides and can regulate the expression of more than 30% of all genes by imperfect base pairing with 3′-untranslated region (3′-UTR) of the target mRNAs at post-transcriptional level [10, 11]. Growing evidences show that abnormal miRNA expression has been detected in almost all human cancers [12, 13] and contributes to tumor initiation, cancer progression and clinical outcome of cancer patients [13, 14], which suggests that miRNAs could be potential targets for cancer therapy, and studies on miRNAs have provided a new possibility for the treatment of cancer.

MiRNAs can either function as oncogenes or tumor suppressors, in accordance with their expression in malignancies and the role in cellular transformation.

Overexpression of oncogenic miRNAs is to be related with transformation, metastasis, increased cell viability and proliferation in many solid malignancies. Some miRNAs have been shown to possess tumor suppressor character, as loss of function of them promotes tumourigenesis [15, 16]. In this regard, therapeutic potentials of RNA oligonucleotides have been proposed as the most direct way for molecules to correct the abnormally expressed miRNAs, including two possible approaches of blocking oncogenic miRNAs using anti-miRNA oligonucleotides or replacing tumor suppressor miRNAs using miRNA mimetics [17].

However, in contrast to some other types of cancer, such as breast cancer and colorectal cancer, little is identified about the function of miRNAs in the pathogenesis of OS. It was found that OS cell lines in general are extremely tumorigenic by evaluating the *in vivo* tumorigenicity, *in vitro* colony-forming potential, invasive/migratory capacity and proliferation ability of 22 OS cell lines. There was a strong association among motility, invasion and colony formation, especially for the exceedingly aggressive OS cell lines, such as HOS-143B. Comparing the miRNA expression profiles of high (such as MG-63, HOS and OSA) and low (such as HAL, IOR/MOS, IOR/OS9, IOR/OS14 and ZK-58) clonogenic OS cell lines discovered that miRNAs were differentially expressed between the two groups. One of them was miR-155-5p, which was highly expressed in all OS cell lines that formed a high number of colonies, and less expressed or absent in OS cell lines having a low clonogenic capability [18]. Tumorigenic and non-tumorigenic OS cell subpopulations also exhibit distinct miRNA expression profiles. A total of 268 miRNAs were identified significantly dysregulated in OS cell line MG-63 compared with the osteoblast cell line HOB [19].

The aim of this meta-analysis is to evaluate the potential value of miRNAs as therapeutic targets for OS based on the published lit­eratures, and to explore the methodological quality of current studies, with the intention to guide the rigour of preclinical experimental design and the future clinical trials.

## RESULTS

### Literature selection

**Figure 1:**
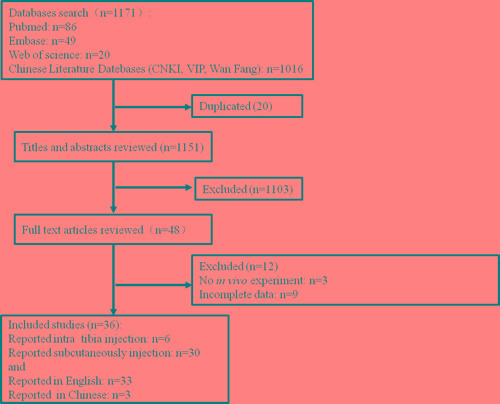
Schematic representation of the literature identification and selection process

The outline of literature selection process is shown in Figure 1. Our database search retrieved 1171 publications following the search strategy described in the section of methods and 20 of the duplicated ones were excluded. After reading the titles and abstracts, 1103 publications were excluded. By a full text review of the 48 publications, 12 studies were further excluded due to they had no *in vivo* experiments [20-22](*n* = 3)or exhibited incomplete data [23-31](*n* = 9). Thirty six of the publications met the criteria were included in the final meta-analysis. Thirty three of the publications were reported in English [17, 25, 32-62], and 3 of them were reported in Chinese [63-65].

### Study characteristics

Among all the 36 included studies, 34 of them used nude mice, while the strain of mice used in 2 studies was not clear. Ten studies used female mice, 9 studies used males, 1 study used female or male mice, and the gender of mice in 16 studies was not presented in the literatures.

Median sample size of mice for the 36 included studies was 16 (range from 8 to 30). The main composition of background diet used in the included studies was not reported. OS xenograft models of the mice used in 30 studies were established by subcutaneous injection, and in 6 studies were established by intratibial injection.

MiRNAs were transfected into human OS cells before inoculating mice ( 29 of 36 included studies) [25, 32-38, 40-42, 44, 45, 48-50, 53-65], injected into the tumor ( 4 of 36 included studies) [39, 46, 52, 58], systemic administrated by tail vain injection(3 of 36 included studies) [17, 43, 47]. The included studies reported the outcomes of tumor weight, tumor volume, or both of them (Table 1).

**Table 1:** Description of the characteristics of studies included in the meta-analysis. (NC=negative control)

Study	Animals	Number of animals	Osteosarcoma xerograph method	miRNA	Experimental groups	Control group	Outcome
Lei Fan 2013[65]	16 female or male BALB/c nude mice	8/8	Subcutaneous	miR-145	MG-63+miR-145	MG-63	Tumor volume Tumor weight
Jie Gao 2012[64]	10 female BALB/c nude mice (4 weeks)	5/5/	Intratibial	miR-195	F5M2+pSilencer 4.1-CMV-miR-195	F5M2+pSilencer 4.1-CMV-NC	Tumor volume Tumor weight
Jie Jin 2013[63]	15 SCID nude mice	5/5/5	Subcutaneous	miR-218	Saos-2+pcDNA3.1-miR-218	A:Saos-2 B:Saos-2+pcDNA3.1-NC	Tumor volume
Fang Ji 2013(a)[40]	8 BALB/c nude mice (4 weeks)	4/4	Subcutaneous	miR-133a	MG-63+miR-133a	MG-63+NC	Tumor volume
Fang Ji 2013(b)[40]	8 BALB/c nude mice (4 weeks)	4/4	Subcutaneous	miR-133a	U2 OS+miR-133a	U2 OS+NC	Tumor volume
Chi Cheng 2014 [44]	12 BALB/c nude mice (4 weeks)	6/6	Subcutaneous	miR-320	U2 OS+miR-320	U2 OS+NC	Tumor volume Tumor weight
Guoxing Xu 2014 [35]	10	5/5	Subcutaneous	miR-142-3p	HOS+pcDNA3.1-miR-142-3p	HOS+pcDNA3.1	Tumor volume Tumor weight
Hao Zhang 2010(a) [41]	12 female BALB/c nude mice (4 weeks)	6/6	Subcutaneous	miR-143	MG-63+miR-143	MG-63+NC	Tumor volume
Hao Zhang 2010(b) [41]	12 female BALB/c nude mice (4 weeks)	6/6	Subcutaneous	miR-143	U2 OS+miR-143	U2 OS+NC	Tumor volume
Tomohiro Fujiwara 2014 [43]	25 Athymic nude mice (5weeks)	5/5/5/5/5	Intratibial	miR-133a	A:143B, LNA-miR-133a/Saline B:143B, LNA-NC/CDDP C:143B, LNA-miR-133a/CDDP Injected via the tail vain	A: 143B, Saline/Saline B:143B, LNA-NC/Saline Injected via the tail vain	Tumor weight
Lei Song 2013[38]	8 female BALB/c nude mice (5-6weeks)	4/4	Subcutaneous	miR-24	MG-63 +lentiviruse-miR-24	MG-63+lentiviruse-NC	Tumor volume
Xinyu Wu 2013(a) [36]	18 female BALB/c nude mice (4-6weeks)	6/6/6	Subcutaneous	miR-34a	MG-63+pcDNA3.1 -miR-34a	A:MG-63 B:MG-63+pcDNA3.1	Tumor volume
Jin Wang 2014 [37]	10 BALB/c nude mice	5/5	Subcutaneous	miR-132	143B+lentiviruse-miR-132	143B+lentiviruse-NC	Tumor volume Tumor weight
Guodong LI 2012 [39]	18 nude mice (4-6weeks)	6/6/6	Subcutaneous	miR-223	MG-63, pcDNA-miR-223 Intratumor injection	A:MG-63,PBS B:MG-63,pcDNA3.1 Intratumor injection	Tumor volume
Lei Chen 2013 [45]	10 male BALB/c nude mice (5weeks)	5/5	Subcutaneous	miR-16	U2 OS +lentiviruse-miR-16	U2 OS+lentiviruse-NC	Tumor volume Tumor weight
Zhengyu Xu 2014[34]	12 BALB/c nude mice	6/6	Subcutaneous	miR-214	Saos-2 +lentiviruse-miR-214	Saos-2+lentiviruse-NC	Tumor volume Tumor weight
Xinyu Wu 2013(b) [36]	18 female BALB/c nude mice (4-6weeks)	6/6/6	Subcutaneous	miR-34a	Saos-2+pcDNA3.1 -miR-34a	A:Saos-2 B:Saos-2+pcDNA3.1	Tumor volume
Kang Yan 2012 [33]	12 female BALB/c nude mice (4weeks)	6/6	Intratibial	miR-34a	SOSP-9607+pcDNA-miR-34a	SOSP-9607+pcDNA3.1	Tumor volume Tumor weight
Mitsuhiko Osaki 2011 [17]	20 male nude mice (5-6weeks)	10/10	Intratibial	miR-143	143B+Luc, miR-143 Injected via the tail vain	143B+Luc, NC Injected via the tail vain	Tumor weight
Kang Han 2014 [42]	30 female BALB/c nude mice (4weeks)	10/10/10	Intratibial	miR-194	SOSP-9607+lentiviruse-miR-194	A:SOSP-9607 B:SOSP-9607+lentiviruse-NC	Tumor volume Tumor weight
Xin Zhou 2013 [32]	12 BALB/c nude mice (5weeks)	6/6	Subcutaneous	miR-340	Saos-2+lentiviruse-miR-340	Saos-2+lentiviruse-NC	Tumor weight
Masanori Kawano 2015[56]	21 BALB/c nude mice (6 weeks)	7/7/7	Subcutaneous	miR-93	Saos-2+miR-93	A:Saos-2+NC B:Untreated	Tumor volume
Yong Zhao 2015 [47]	12 male athymic nude mice (4–6-weeks)	6/6	Subcutaneous	miR-34a	143B, miR-34a Injected via tail vein	Vehicle Injected via tail vein	Tumor volume Tumor weight
K Tian 2015 [51]	30 C57BL/6 mice (8 weeks)	10/10/10	Subcutaneous	miR-23a	HOS58+ pGL3-miR23a-EGFP	A:HOS58 B: HOS58+ pGL3-Ctrl-EGFP	Tumor volume Tumor weight
Guoqing Duan 2015 [60]	14 female BALB/c nude mice (6 weeks)	7/7	Subcutaneous	miR-26b	U2OS+ pcDNA3.1-miR-26b	U2OS+pcDNA3.1- anti-miR-26b	Tumor volume
Jiahui Zhou (a)2015[46]	18 BALB/c nude mice(20g)	9/9	Subcutaneous	miR-143	Saos-2, AdmiR-143 intratumorally	Saos-2, ADNC intratumorally	Tumor weight
Jiahui Zhou (b) 2015 [46]	18 BALB/c nude mice(20g)	9/9	Subcutaneous	miR-143	U2OS, AdmiR-143 intratumorally	U2OS, ADNC intratumorally	Tumor weight
Wei Wang 2015[61]	12 BALB/c nude mice (4 weeks)	6/6	Subcutaneous	miR-144	143B+ lentiviruse-miR-144	143B+ lentiviruse-NC	Tumor volume Tumor weight
Xiaoji Luo 2014[54]	10 male BALB/c nude mice (4 weeks)	5/5	Subcutaneous	miR-212	MG-63+ miR-212	MG-63+NC	Tumor volume Tumor weight
Xuming Wang 2014 [50]	8 BALB/c nude mice (4-6 weeks)	4/4	Subcutaneous	miR-214	Saos-2+pcDNA3.1-miR-214	Saos-2+pcDNA3.1	Tumor volume Tumor weight
Wei Liu 2015 [55]	10 BALB/c nude mice(6 weeks)	5/5	Subcutaneous	miR-49 0-3p	Saos-2+ miR-49 0-3p	Saos-2+NC	Tumor volume Tumor weight
Liang Ge 2016[59]	20 male BALB/c mice(5-6 weeks)	10/10	Subcutaneous	miR-497	MG-63+ miR-497	MG-63+NC	Tumor volume Tumor weight
Xiuhui Wang 2014[49]	12 male BALB/c nude mice (4 weeks)	6/6	Subcutaneous	miR-25	Saos-2+ miR-25	Saos-2+NC	Tumor weight
Yu He 2014[58]	16 male BALB/c nude mice (5 weeks)	8/8	Subcutaneous	miR-23a	MG-63, miR-23a intratumorally	MG-63, NC	Tumor volume Tumor weight
Xiaohui Sun 2015[52]	12 male BALB/c nude mice	6/6	Subcutaneous	miR-155	U2OS, anti-miR-155 intratumorally	U2OS, anti-NC intratumorally	Tumor volume Tumor weight
Zhengwen Sun 2014[25]	10 male BALB/c nude mice (4 weeks)	5/5	Subcutaneous	miR-202	HOS+ lentiviruse-miR-202	HOS+ lentiviruse-NC	Tumor volume Tumor weight
Meng Xu 2014[48]	16 female athymic nude mice(6 weeks)	8/8	Subcutaneous	miR-382	CD133high OS primary tumor cell+miR-382	CD133high OS primary tumor cell+NC	Tumor volume
Baoyong Sun 2015[53]	16 female BALB/c athymic nude mice (3–4 weeks)	8/8	Subcutaneous	miR-217	MG-63+ lentiviruse -miR-217	MG-63+ lentiviruse-NC	Tumor volume Tumor weight
Tatsuya Iwasaki 2015[57]	21 nude mice	7/7/7	Subcutaneous	miR-let-7a	MG-63+ miR-let-7a	A: MG-63 B: MG-63+NC	Tumor volume
Kang Han 2015[62]	20 female BALB/c nude mice(4 weeks)	10/10	Intratibial	miR-195	SOSP-9607+ lentiviruse -miR-195	SOSP-9607+ lentiviruse -NC	Tumor volume Tumor weight

### Quality assessments of the included experiments

The quality assessment of each included publication in this meta-analysis is shown in Table 2. No studies included here described allocation concealment and sample-size calculation, reported animals excluded from analysis or blinded assessment of outcome. One study reported inclusion and exclusion criteria [39]. Six studies reported randomization [39, 46, 47, 55, 58, 65], whereas 23 studies reported potential conflicts of interest and study funding [25, 33, 36, 37, 39, 40, 43, 44, 46-48, 50, 52-58, 60-62, 64]. Only one study reported blinded assessment of outcome [53]. Therefore, the methodological quality of studies included here was not satisfied.

**Table 2:** Quality assessment of the included experiments

Study	Sample-size calculation	Inclusion and exclusion criteria	Randomization	Allocation concealment	Reporting of animals excluded from analysis	Blinded assessment of outcome	Reporting potential conflicts of interest and study funding
Lei Fan 2013 [65]	Unclear	Unclear	Yes	Unclear	Unclear	Unclear	Unclear
Jie Gao 2012 [64]	Unclear	Unclear	Unclear	Unclear	Unclear	Unclear	Yes
Jie Jin 2013 [63]	Unclear	Unclear	Unclear	Unclear	Unclear	Unclear	Unclear
Fang Ji 2013[40]	Unclear	Unclear	Unclear	Unclear	Unclear	Unclear	Yes
Chi Cheng 2014 [44]	Unclear	Unclear	Unclear	Unclear	Unclear	Unclear	Yes
Tomohiro Fujiwara 2014 [43]	Unclear	Unclear	Unclear	Unclear	Unclear	Unclear	Yes
Hao Zhang 2010 [41]	Unclear	Unclear	Unclear	Unclear	Unclear	Unclear	Unclear
Lei Song 2013 [38]	Unclear	Unclear	Unclear	Unclear	Unclear	Unclear	Unclear
Xinyu Wu 2013 [36]	Unclear	Unclear	Unclear	Unclear	Unclear	Unclear	Yes
Kang Yan 2012[33]	Unclear	Unclear	Unclear	Unclear	Unclear	Unclear	Yes
Mitsuhiko Osaki 2011[17]	Unclear	Unclear	Unclear	Unclear	Unclear	Unclear	Unclear
Kang Han 2014 [42]	Unclear	Unclear	Unclear	Unclear	Unclear	Unclear	Unclear
Xin Zhou 2013 [32]	Unclear	Unclear	Unclear	Unclear	Unclear	Unclear	Unclear
Zhengyu Xu 2014[34]	Unclear	Unclear	Unclear	Unclear	Unclear	Unclear	Unclear
Lei Chen 2013[45]	Unclear	Unclear	Unclear	Unclear	Unclear	Unclear	Unclear
Jin Wang 2014 [37]	Unclear	Unclear	Unclear	Unclear	Unclear	Unclear	Yes
Guoxing Xu 2014[35]	Unclear	Unclear	Unclear	Unclear	Unclear	Unclear	Unclear
Guodong LI 2013 [39]	Unclear	Yes	Yes	Unclear	Unclear	Unclear	Yes
Masanori Kawano 2015[56]	Unclear	Unclear	Unclear	Unclear	Unclear	Unclear	Yes
Yong Zhao 2015[47]	Unclear	Unclear	Yes	Unclear	Unclear	Unclear	Yes
K Tian 2015[51]	Unclear	Unclear	Unclear	Unclear	Unclear	Unclear	Unclear
Guoqing Duan 2015[60]	Unclear	Unclear	Unclear	Unclear	Unclear	Unclear	Yes
Jiahui Zhou 2015[46]	Unclear	Unclear	Yes	Unclear	Unclear	Unclear	Yes
Wei Wang 2015[61]	Unclear	Unclear	Unclear	Unclear	Unclear	Unclear	Yes
Xiaoji Luo 2014[54]	Unclear	Unclear	Unclear	Unclear	Unclear	Unclear	Yes
Xuming Wang 2014[50]	Unclear	Unclear	Unclear	Unclear	Unclear	Unclear	Yes
Wei Liu 2015[55]	Unclear	Unclear	Yes	Unclear	Unclear	Unclear	Yes
Liang Ge 2016[59]	Unclear	Unclear	Unclear	Unclear	Unclear	Unclear	Unclear
Xiuhui Wang 2014[49]	Unclear	Unclear	Unclear	Unclear	Unclear	Unclear	Unclear
Yu He 2014[58]	Unclear	Unclear	Yes	Unclear	Unclear	Unclear	Yes
Xiaohui Sun 2015[52]	Unclear	Unclear	Unclear	Unclear	Unclear	Unclear	Yes
Zhengwen Sun 2014[25]	Unclear	Unclear	Unclear	Unclear	Unclear	Unclear	Yes
Meng Xu 2014[48]	Unclear	Unclear	Unclear	Unclear	Unclear	Unclear	Yes
Baoyong Sun 2015[53]	Unclear	Unclear	Unclear	Unclear	Unclear	Yes	Yes
Tatsuya Iwasaki 2015[57]	Unclear	Unclear	Unclear	Unclear	Unclear	Unclear	Yes
Kang Han 2015[62]	Unclear	Unclear	Unclear	Unclear	Unclear	Unclear	Yes

### Inhibitory effects on the tumor growth (tumor weight/ tumor volume) of osteosarcoma xenograft models by correction of the abnormally expressed miRNAs

Due to the data used for this systematic review and meta-analysis were experiment-levels, different major outcome measures (tumor weight or tumor volume); different types(miR-195, miR-143, miR-34a, miR-214, miR-23a, miR-133a, and so on) or functions(oncogenes or tumor suppressors) of miRNAs; different miRNA intervention methods(directly transfected into OS cells, transfected into OS cells with plasmid vectors, infected into OS cells by lentivirus vectors, systematic administration or injected into tumor directly); different OS cells(MG-63, U2 OS, Saos-2,143B or SOSP-9607) and inoculation sites for producing xenograft models (intratibial inoculation or subcutaneous inoculation) were used in the included studies, these factors all could potentially produce a high heterogeneity. However, if all the baseline characteristics among groups were balanced, the data could not be evaluated. Therefore, stratifications were performed based on these factors to minimize the heterogeneity. Meanwhile, random-effects models were used for the analyses.

#### When all the included studies used tumor weight as the major outcome measure were stratified by whether the aberrantly expressed miRNAs function as oncogenes or tumor suppressors in the pathogenesis of osteosarcoma

Since the mechanisms of oncogenes or tumor suppressors in the pathogenesis of OS are different, we conducted the delaminating analysis.

**Figure 2:**
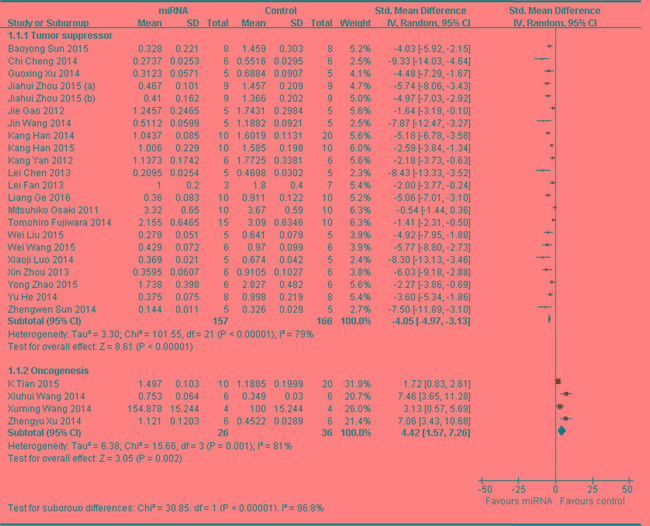
Meta-analysis of studies evaluating the inhibitory effects on tumor weight after the aberrantly expressed miRNAs were corrected, when all included studies used tumor weight as the major outcome measure were stratified by the function of miRNAs in the pathogenesis of osteosarcoma SD, standard deviation; CI, confidence interval.

Twenty-one of the 25 studies reported that miRNAs function as the tumor suppressors and thus the data were combined for a meta-analysis [17, 25, 32, 33, 35, 37, 42-47, 53-55, 58, 59, 61, 62, 64, 65]. A total of 157 mice in the intervention arm and 166 in the control arm were included. The results suggested that restoring the decreased tumor suppressor miRNAs was able to restrain the progression of OS *in vivo* when a random-effects model was used. And the pooled MD = [ -4.05]; 95% confidence interval [CI]: [-4.97]- [-3.13]; *p* < 0.00001(Figure 2 upper part).

Four of the 25 studies reported that miRNAs functions as onco-miRNAs in OS [34, 49-51]. A total of 26 mice in the intervention arm and 36 in the control arm were included. The results suggested that decreasing the tumor onco-miRNAs was also able to restrain the OS progression *in vivo* when a random-effects model was used. And the pooled MD = [4.42]; 95% confidence interval [CI]: [1.57]- [7.26]; *p* = 0.001; Figure 2, lower part.

#### When above included studies that reported miRNAs as tumor suppressors or oncogenes were further stratified respectively by the following factors

##### The miRNA delivery method

**Figure 3:**
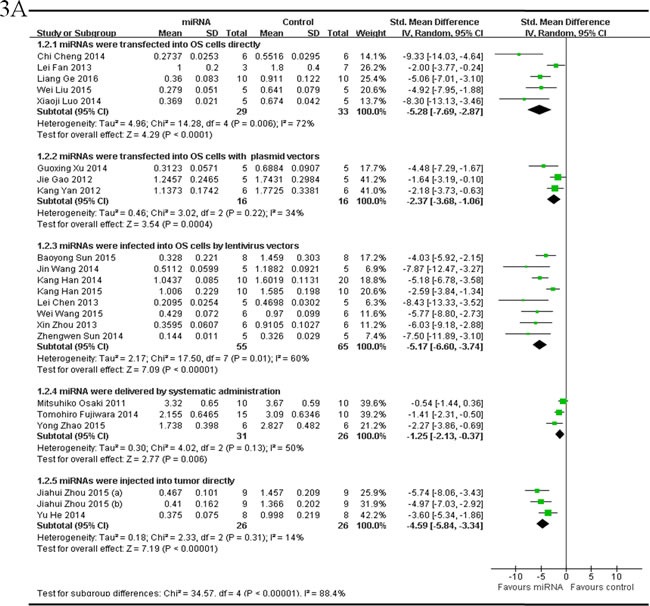

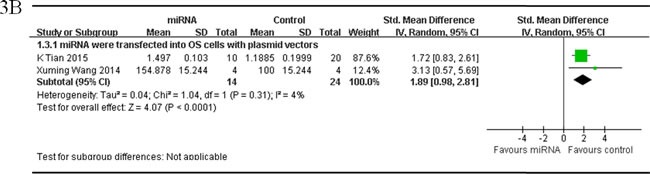
Meta-analysis of included studies evaluating the inhibitory effects on tumor weight after the aberrantly expressed miRNAs were corrected, when studies, reported miRNAs as tumor suppressors **A.** or oncogenes **B.** and used tumor weight as the major outcome measure, were stratified respectively by the miRNA delivery method. SD, standard deviation; CI, confidence interval.

There were 21 studies which reported that tumor weight as the major outcome measure and miRNAs as tumor suppressors. MiRNAs were directly transfected into the OS cells in 5 studies, and thus the data were combined for a meta-analysis [44, 54, 55, 59, 65]. There were 29 mice in the intervention arm and 33 mice in the control arm. The tumor weight showed a significant statistical difference when the decreased tumor suppressor miRNAs were corrected (pooled MD = [-5.28]; 95% confidence interval [CI]: [-7.69]- [-2.87]; *p* = 0.006; Figure 3A, part 1) in a random-effects model. MiRNAs with plasmid vectors were transfected into the OS cells in 3 studies, and were combined for a meta-analysis. There were 16 mice in both the intervention and control arms. Tumor weight was also significantly inhibited after the correction of the decreased tumor suppressor miRNAs (pooled MD = [-2.37]; 95% confidence interval [CI]: [-3.68]- [1.06]; *p* = 0.22; Figure 3A, part 2) in a random-effects model [33, 35, 64]. MiRNAs were infected into the OS cells by lentivirus vectors in 8 studies, and were combined for a meta-analysis. There were 55 mice in the intervention arm and 65 in the control arm. The tumor weight significantly decreased when the decreased tumor suppressor miRNAs were corrected (pooled MD = [-5.17]; 95% confidence interval [CI]: [-6.60]- [-3.74]; *p* = 0.01; Figure 3A, part 3)in a random-effects model [25, 32, 37, 42, 45, 53, 61, 62]. MiRNAs were delivered by systematic administration after inoculation with OS cells in other 3 studies [17, 43, 47]. There were 31 mice in the intervention arm and 26 mice in the control arm. Tumor weight also showed a statistically significant decrease after the aberrantly expressed miRNAs were corrected (pooled MD = [-1.25]; 95% confidence interval [CI]: [-2.13]- [-0.37]; *p* = 0.13; Figure 3A, part 4) in a random-effects model. MiRNAs were injected into tumor directly in 2 studies, and were combined for a meta-analysis. There were 26 mice in both of the intervention and control arms. The tumor weight significantly decreased when the aberrantly expressed miRNAs were corrected (pooled MD = [-4.59]; 95% confidence interval [CI]: [-5.84]- [-3.34]; *p* = 0.31; Figure 3A, part 5)in a random-effects model [46, 58].

There were 4 studies which reported that tumor weight as the major outcome measure and miRNAs as oncogenes. MiRNAs were transfected into OS cells with plasmid vectors in 2 studies, and the data were combined for a meta-analysis. There were 14 mice in the intervention arm and 24 in the control arm. Tumor weight was also significantly inhibited after correction of the up-regulated oncogene miRNAs (pooled MD = [1.89]; 95% confidence interval [CI]: [0.98]- [2.81]; *p* = 0.31; Figure 3B) in a random-effects model [50, 51].

Only 1 study respectively reported that miRNA was either directly transfected into the OS cells or infected into the OS cells by lentivirus vectors, therefore the data could not be pooled. One of the 2 studies reported that miR-25 was transfected into OS cells directly [49]. This study confirmed that miR-25 was frequently over-expressed in OS, and up-regulation of miR-25 promoted cell proliferation *in vitro* and tumor growth in a xenograftmouse model. The other study reported that miR-214 was infected into OS cells by lentivirus vectors [34]. This study showed that miR-214 was frequently up-regulated in OS specimens than noncancerous, and over-expression of miR-214 could promote OS cell proliferation, invasion and tumor growth in nude mice. The weight of miR-214-overexpressing tumor was > 2-fold higher than that of the controls.

As we could see in Figure 3A, the overall effect on reducing the tumor weight *via* miRNAs being infected into OS cells with lentivirus vectors or transfected into OS cells directly were the best and comparable, then direct injecting miRNAs into tumors, and followed by being transfected into OS cells with plasmid vectors or systematic administration of miRNAs.

##### The names of miRNAs

**Figure 4:**
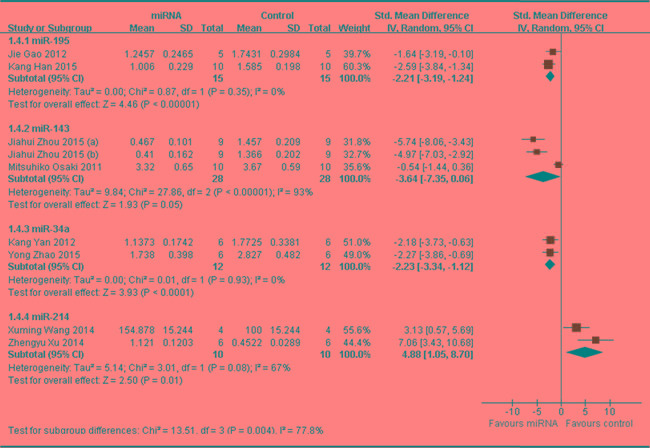
Meta-analysis of included studies evaluating the inhibitory effects on tumor weight after the aberrantly expressed miRNAs were corrected, when all included studies used tumor weight as the major outcome measure were stratified by the names of miRNAs SD, standard deviation; CI, confidence interval.

In order to find out if different miRNA has different influence on OS growth, data of same miRNA from more than 2 studies (if have), which reported that tumor weight was the major outcome measure, were combined together for a meta-analysis. This resulted 4 different miRNAs were anylyzed, including 3 tumor suppressor miRNAs(miR-195, miR-143 and miR-34a) and 1 oncogene(miR-214). As shown in Figure 4, part 1, there were 15 mice in both the interventionand the control arms. Tumor weight significantly decreased when the expression of down-regulated miR-195 was recovered (pooled MD = [ -2.21]; 95% confidence interval [CI]: [-3.19]- [-1.24]; *p* = 0.35;) [62, 64]; there were 28 mice in the intervention arm and 28 in the control arm. Tumor weight significantly decreased when the expression of down-regulated miR-143 was recovered (pooled MD = [ -3.64]; 95% confidence interval [CI]: [-7.35]- [0.06]; *p* < 0.00001; Figure 4, part 2) [17, 46]; there were 12 mice in the intervention arm and 12 in the control arm. Tumor weight significantly decreased when the expression of down-regulated miR-34a was rescued (pooled MD = [ -2.23]; 95% confidence interval [CI]: [-3.34]- [-1.12]; *p* = 0.93; Figure 4, part 3) [33, 47]; there were 10 mice in the intervention arm and 10 in the control arm. Tumor weight significantly decreased when the expression of up-regulated miR-214 was down-regulated (pooled MD = [4.88]; 95% confidence interval [CI]: [1.05]- [8.70]; *p* = 0.08; Figure 4, part 4) [34, 50].

As we could see in Figure 4A, the effect on inhibiting tumor weight was most significant when the aberrantly expressed oncogene miR-214 was corrected, and followed by rescuing miR-143, then miR-195 or miR-34a. The efficacy due to up-regulating miR-195 or miR-34a was comparable.

##### Inoculation sites of osteosarcoma cells

There were 21 studies which reported that tumor weight as the major outcome measure and miRNAs as tumor suppressors.

**Figure 5:**
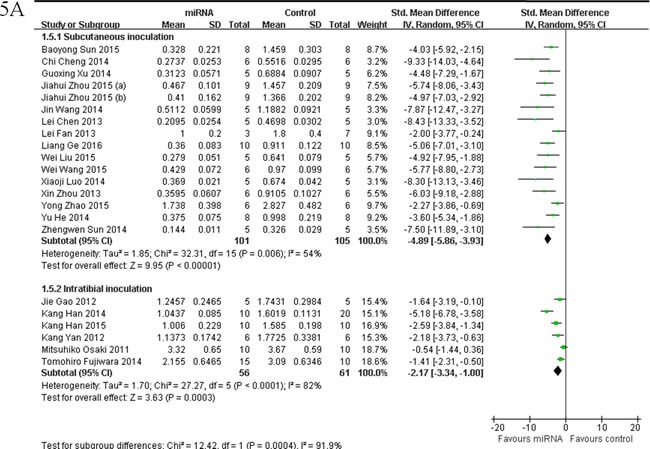

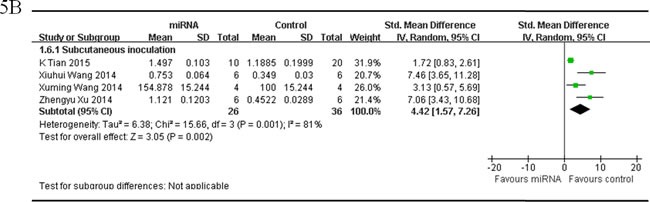
Meta-analysis of included studies evaluating the inhibitory effects on tumor weight after the aberrantly expressed miRNAs were corrected, when studies reported miRNAs as tumor suppressors **A.** or oncogenes **B.** used tumor weight as the major outcome measure were stratified by injection sites of osteosarcoma cells. SD, standard deviation; CI, confidence interval.

Fifteen of them applied OS xenograft models produced by subcutaneous inoculation of OS cells [25, 32, 35, 37, 44-47, 53-55, 58, 59, 61, 65]. These 15 studies were combined together for the meta-analysis, and there were 101 mice in the intervention arm and 105 in the control arm. Given that the heterogeneity was high among the studies, a random-effects model was selected, and the tumor weight considerably decreased when the decreased tumor suppressor miRNAs were corrected (pooled MD = [-4.89]; 95% confidence interval [CI]: [-5.86]- [-3.93]; *p* = 0.006; Figure 5A, upper part).

OS xenograft models induced by intratibial injection of OS cells were used in other 6 studies [17, 33, 42, 43, 62, 64]. Fifty-six mice in the intervention arm and 61 mice in the control arm were included. A random-effects model was used also due to the high heterogeneity among the included studies, and the tumor weight significantly decreased after the decreased tumor suppressor miRNAs were corrected (pooled MD = [ -2.17]; 95% confidence interval [CI]: [-3.34]- [-1.00]; *p* <0.0001; Figure 5A, lower part).

As we could see in Figure 5A, the overall effects on reducing the tumor weight were more significant when the OS xenograft models were produced by subcutaneous injection than by intratibial injection.

There were 4 studies reported tumor weight as the major outcome measure and miRNAs as oncogenes, which used OS xenograft models produced by subcutaneous inoculation of OS cells [34, 49-51] . There were 26 mice in the intervention arm and 36 in the control arm. Given that the heterogeneity was high among the studies, a random-effects model was selected, and the tumor weight considerably decreased when the expressions of the oncogene miRNAs were corrected (pooled MD = [4.42]; 95% confidence interval [CI]: [1.57]- [7.26]; *p* = 0.001; Figure 5B).

##### Inoculated osteosarcoma cell lines

**Figure 6:**
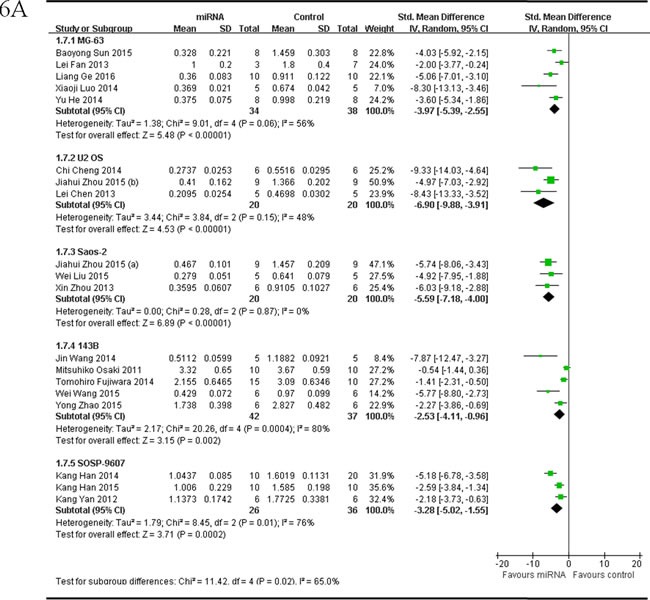

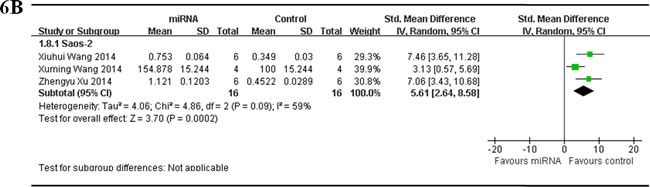
Meta-analysis of included studies evaluating the inhibitory effects on tumor weight after the aberrantly expressed miRNAs were corrected, when studies reported miRNAs as tumor suppressors **A.** or oncogenes **B.** and used tumor weight as the major outcome measure, were stratified by osteosarcoma cell lines used to produce osteosarcoma xenograft models . SD, standard deviation; CI, confidence interval.

The delaminating analysis based on the 5 different OS cell lines that were used to produce OS xenograft models in the included studies, were performed. Given that the heterogeneity was high across the studies, a random-effects model was chosen. Among studies reported tumor weight as the major outcome measure and miRNAs as tumor suppressors, 5 studies [53, 54, 58, 59, 65], used MG-63 for OS xenograft model, were combined together for the meta-analysis, and there were 34 mice in the intervention and 38 mice in the control arm. Tumor weight significantly decreased when the aberrantly expressed miRNAs were corrected (pooled MD = [ -3.97]; 95% confidence interval [CI]: [-5.39]- [-2.55]; *p* = 0.06; Figure 6A, part 1);data from the 3 studies [44-46] used U2 OS for OS xenograft model, were combined together for the meta-analysis, and there were 20 mice in both the intervention and control arms. Tumor weight significantly decreased when the aberrantly expressed miRNAs were corrected (pooled MD = [-6.90]; 95% confidence interval [CI]: [-9.88]- [-3.91]; *p* = 0.15; Figure 6A,part 2);data from the 3 studies [32, 46, 55] used Saos-2 for OS xenograft model, were combined together for the meta-analysis, and there were 20 mice in both the intervention and control arms. Tumor weight significantly decreased when the aberrantly expressed miRNAs were corrected (pooled MD = [-5.59]; 95% confidence interval [CI]: [-7.18]- [-4.00]; *p* = 0.87; Figure 6A, part 3); data from the 5 studies [17, 37, 43, 47, 61] used 143B for OS xenograft model, were combined together for the meta-analysis, and there were 42 mice in the intervention arm and 37 in the control arm. Tumor weight noticeably decreased when the aberrantly expressed miRNAs were corrected (pooled MD = [-2.53]; 95% confidence interval [CI]: [-4.11]- [-0.96]; *p*= 0.0004; Figure 6A, part 4); data from the 3 studies [33, 42, 62] used SOSP-9607 for OS xenograft model, were combined together for the meta-analysis, and there were 26 mice in the intervention arm and 36 in the control arm. Tumor weight significantly decreased when the aberrantly expressed miRNAs were corrected (pooled MD = [-3.28]; 95% confidence interval [CI]: [-5.02]- [-1.55]; *p* = 0.01; Figure 6A, part 5).

As we could see in Figure 6A, the overall effects on reducing the tumor weight were most significant when the OS xenograft models were produced by injection of U2 OS cells, then by injection of Saos-2, and followed by injection of MG-63, then143B or SOSP-9607.

There were 3 studies reported tumor weight as the major outcome measure and miRNAs as oncogenes, used Saos-2 for OS xenograft model, were combined together for the meta-analysis, and there were 16 mice in both the intervention and control arms [34, 49, 50]. Tumor weight significantly decreased when the aberrantly expressed miRNAs were corrected (pooled MD = [5.61]; 95% confidence interval [CI]: [2.64]- [8.58]; *p* = 0.09; Figure 6B).

### When all the included studies used tumor volume as the major outcome measure were stratified by whether the abnormally expressed miRNAs function as oncogenes or tumor suppressors in the pathogenesis of osteosarcoma

Thirty-one studies that included measurements of tumor volume were divided into 2 subgroups according to the function of abnormally expressed miRNAs.

**Figure 7:**
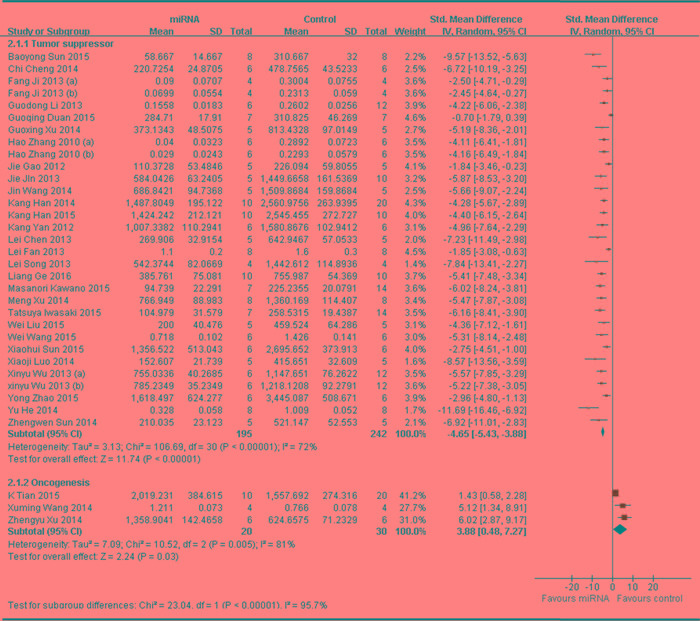
Meta-analysis of studies evaluating the inhibitory effects on tumor volume after the aberrantly expressed miRNAs were corrected, when all included studies used tumor volume as the major outcome measure were stratified by the function of miRNAs in the pathogenesis of osteosarcoma SD, standard deviation; CI, confidence interval.

One subgroup included 28 studies, which reported that miRNAs function as the tumor suppressors, and thus the data were combined for a meta-analysis [25, 33, 35-42, 44, 45, 47, 48, 52-65] A total of 195 mice in the intervention arm and 242 in the control arm were included. The tumor volume was considerably suppressed after the decreased miRNAs were restored in a random-effects model. And the pooled MD = [ -4.65]; 95% confidence interval [CI]: [-5.43]- [-3.88]; *p* < 0.00001; Figure 7, upper part).

The 3 studies reported that miRNAs function as oncogenes in OS as described above, and thus the data were combined for a meta-analysis [34, 50, 51]. A total of 20 mice in the intervention arm and 30 in the control arm were included. The tumor volume was considerably suppressed after the aberrantly expressed miRNAs were restored in a random-effects model. And the pooled MD = [3.88]; 95% confidence interval [CI]: [0.48]- [7.27]; *p* = 0.005 ( Figure 7, lower part).

#### When above included studies that reported miRNAs as tumor suppressors or oncogenes were further stratified respectively by the following factors

##### The miRNA delivery method

**Figure 8:**
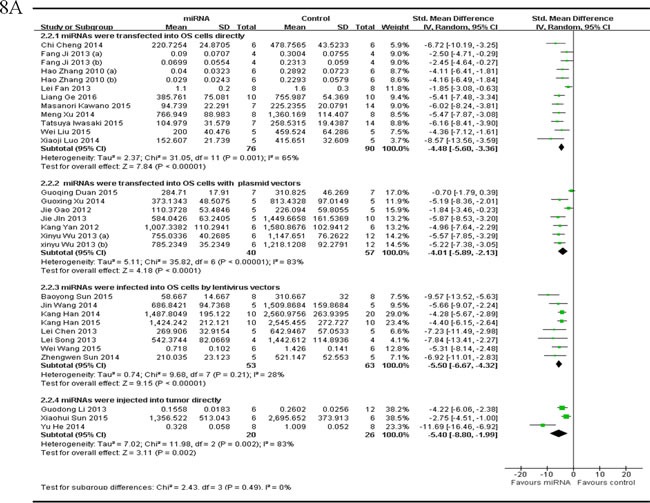

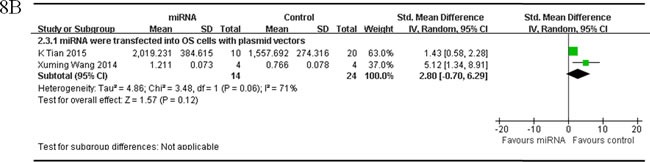
Meta-analysis of included studies evaluating the inhibitory effects on tumor volume after the aberrantly expressed miRNAs were corrected, when studies reported miRNAs as tumor suppressors **A.** or oncogenes **B.** and used tumor volume as the major outcome measure were stratified by the miRNA delivery method. SD, standard deviation; CI, confidence interval.

There were 28 studies which reported that tumor volume as the major outcome measure and miRNAs as tumor suppressors. MiRNAs were directly transfected into the OS cells in 10 studies, and thus the data were combined for a meta-analysis [40, 41, 44, 48, 54-57, 59, 65] There were 76 mice in the intervention arm and 90 mice in the control arm. The tumor volume showed a statistically significant difference when the decreased miRNAs were corrected (pooled MD = [-4.48]; 95% confidence interval [CI]: [-5.60]- [-3.36]; *p* = 0.001; Figure 8A, part 1) in a random-effects model. MiRNAs with vectors of plasmids were transfected into the OS cells in 6 studies, and were combined for a meta-analysis. There were 40 mice in the intervention arm and 57 in the control arm. The tumor volume significantly reduced when the decreased tumor suppressor miRNAs were corrected (pooled MD = [ -4.01]; 95% confidence interval [CI]: [-5.89]- [-2.13]; *p* < 0.00001; Figure 8A, part 2)in a random-effects model [33, 35, 36, 60, 63, 64]. MiRNAs were infected into the OS cells by lentivirus vectors in 8 studies, and were combined for a meta-analysis. There were 53 mice in the intervention arm and 63 in the control arm. The tumor volume significantly decreased when the decreased tumor suppressor miRNAs were corrected (pooled MD = [-5.50]; 95% confidence interval [CI]: [-6.67]- [-4.32]; *p* = 0.21; Figure 8A, part 3)in a random-effects model [25, 37, 38, 42, 45, 53, 61, 62]. MiRNAs were injected into tumor directly in 3 studies, and were combined for a meta-analysis. There were 20 mice in the intervention arm and 26 in the control arm. The tumor volume significantly decreased when the aberrantly expressed miRNAs were corrected (pooled MD = [-5.40]; 95% confidence interval [CI]: [-8.80]- [-1.99]; *p* = 0.002; Figure 8A, part 4) in a random-effects model [39, 52, 58].

Only one study reported that miRNA was delivered by the tail vain [47].

As we could see in Figure 8A, the overall effects on reducing the tumor volume showed no significant difference among different miRNA delivery methods, with a slight better of miRNAs being infected with lentivirus vectors or injected into tumor directly, then being directly transfected into OS cells or transfected with plasmid vectors.

There were 3 studies which reported that tumor volume as the major outcome measure and miRNAs as oncogenes. MiRNAs were transfected into OS cells with plasmid vectors in 2 studies, and the data were combined for a meta-analysis [49, 51]. There were 14 mice in the intervention arm and 24 in the control arm. Tumor volume was also significantly inhibited after correction of the oncogene miRNA expression (pooled MD = [2.80]; 95% confidence interval [CI]: [-0.70]- [6.29]; *p* = 0.06; Figure 8B) in a random-effects model.

Only 1 study reported that miRNA was infected into the OS cells by lentivirus vectors as being described above [34].

##### The names of miRNAs

**Figure 9:**
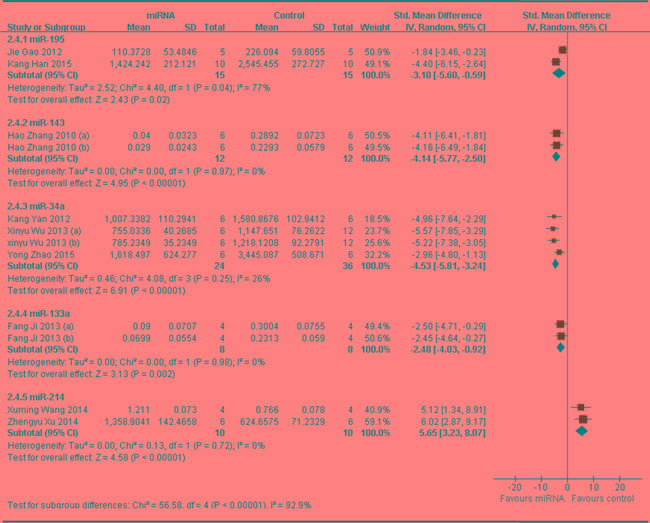
Meta-analysis of included studies evaluating the inhibitory effects on tumor volume after the aberrantly expressed miRNAs were corrected, when all included studies used tumor volume as the major outcome measure were stratified by the names of miRNAs SD, standard deviation; CI, confidence interval.

In order to find out if different miRNA has different influence on OS growth, data of same miRNA from more than 2 studies (if have), which reported that tumor volume was the major outcome measure, were combined together for a meta-analysis. This resulted 5 different miRNAs were analyzed, including 4 tumor suppressor miRNAs(miR-195, miR-143, miR-34a and miR-133) and 1oncogene(miR-214).There were 15 mice in the intervention arm and 15 in the control arm. Tumor volume significantly decreased when the expression of down-regulated miR-195 was recovered (pooled MD = [ -3.10]; 95% confidence interval [CI]: [-5.60]- [-0.59]; *p* = 0.04; Figure 9, part 1) [62, 64]. There were 12 mice in the intervention arm and 12 in the control arm. Tumor volume significantly decreased when the expression of down-regulated miR-143 was recovered (pooled MD = [-4.14]; 95% confidence interval [CI]: [-5.77]- [-2.50]; *p*= 0.97; Figure 9, part 2) [41] .There were 24 mice in the intervention arm and 36 in the control arm. Tumor volume significantly decreased when the expression of down-regulated miR-34a was recovered (pooled MD = [ -4.53]; 95% confidence interval [CI]: [-5.81]- [-3.24]; *p* = 0.25; Figure 9, part 3) [33, 36, 47].There were 8 mice in the intervention arm and 8 in the control arm. Tumor volume there no significantly decreased when the miR-133a was recovered (pooled MD = [-2.48]; 95% confidence interval [CI]: [-4.03]- [-0.92]; *p* = 0.92; Figure 9, part 4) [40].There were 10 mice in the intervention arm and 10 in the control arm. Tumor volume significantly decreased when the expression of up-regulated miR-214 was recovered (pooled MD = [5.65]; 95% confidence interval [CI]: [3.23]- [8.07]; *p* = 0.72; Figure 9, part 5) [34, 50].

As we could see in Figure 9, the effects on inhibiting tumor volume were most significant when the aberrantly expressed miR-34a, miR-143 and miR-214 were corrected, and then followed by miR-195 and miR-133a

##### Inoculation sites of osteosarcoma cells

28 studies reported tumor volume as the major outcome measure and miRNAs as tumor suppressors, were divided into 2 subgroups according to the inoculation sites of OS cells.

**Figure 10:**
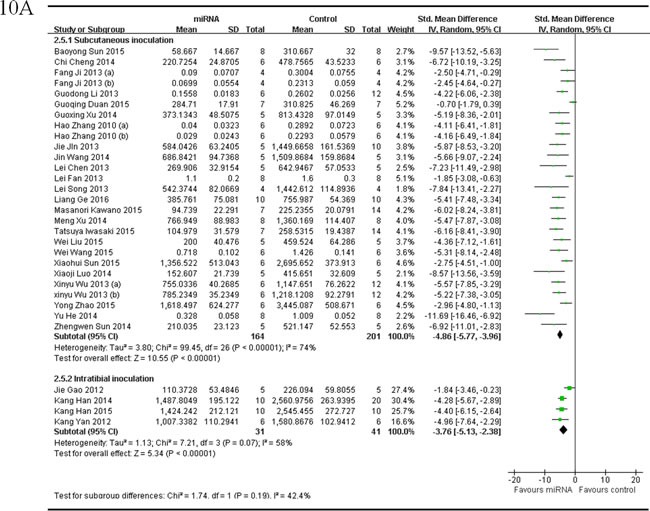

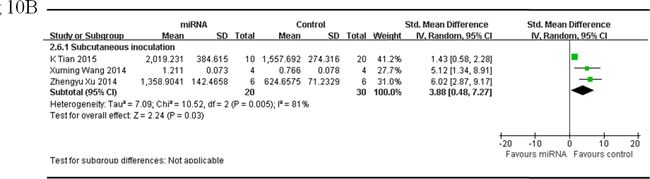
Meta-analysis of included studies evaluating the inhibitory effects on tumor volume after the aberrantly expressed miRNAs were corrected, when studies reported miRNAs as tumor suppressors **A.** or oncogenes **B.** and used tumor volume as the major outcome measure were stratified by injection sites of osteosarcoma cells. SD, standard deviation; CI, confidence interval.

One subgroup included 24 studies that compared the anti-osteosarcoma effects in OS xenograft models produced by subcutaneous injection, with the rectification of the abnormally expressed miRNAs [25, 35-41, 44, 45, 47, 48, 52-61, 63, 65]. There were164 mice in the intervention arm and 201 in the control arm. The tumor volume was significantly suppressed by correcting the abnormally expressed miRNAs (pooled MD = [-4.86]; 95% confidence interval [CI]: [-5.77- [-3.96]; *p* < 0.00001; Figure 10A, upper part) in a random-effects model.

Another subgroup had 4 studies that compared the anti-osteosarcoma effects in OS xenograft models produced by intratibial injection, with the rectification of the abnormally expressed tumor suppressor miRNAs [33, 42, 62, 64]. There were 31 mice in the intervention arm and 41 mice in the control arm. The tumor volume significantly decreased by correcting the abnormally expressed miRNAs (pooled MD = [-3.76]; 95% confidence interval [CI]: [-5.13]- [-2.38]; *p* = 0.07; Figure 10A, lower part) in a random-effects model.

As shown in Figure 10A, the overall effects on inhibiting tumor volume were better when the OS xenograft models were produced by subcutaneous injection than by intratibial injection.

There were 3 studies reported tumor volume as the major outcome measure and miRNAs as oncogenes, which used OS xenograft models produced by subcutaneous inoculation of OS cells [34, 50, 51]. There were 20 mice in the intervention arm and 30 in the control arm. Given that the heterogeneity was high among the studies, a random-effects model was selected, and the tumor weight considerably decreased when the expressions of the oncogene miRNAs were corrected (pooled MD = [3.88]; 95% confidence interval [CI]: [0.48]- [7.27]; *p* = 0.005; Figure 10B).

##### Inoculated osteosarcoma cell lines

**Figure 11:**
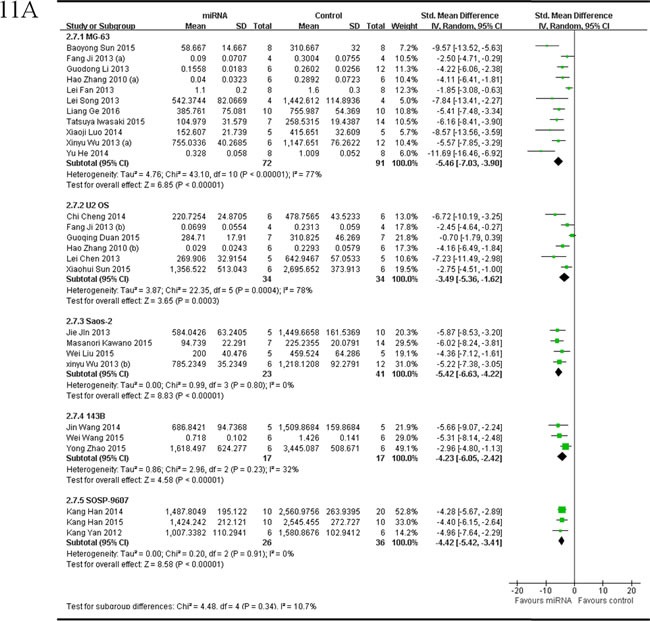

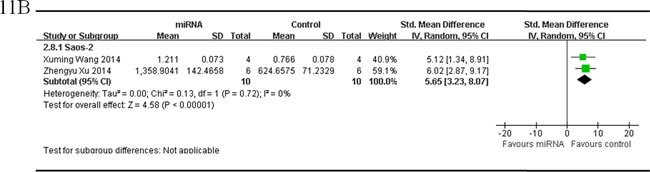
Meta-analysis of included studies evaluating the inhibitory effects on tumor volume after the aberrantly expressed miRNAs were corrected, when studies reported miRNAs as tumor suppressors **A.** or oncogenes **B.** and used tumor volume as the major outcome measure were stratified by osteosarcoma cell lines used to produce OS xenograft models. SD, standard deviation; CI, confidence interval.

The delaminating analysis of data from studies reported tumor volume as the major outcome measure was performed, based on the 5 different OS cell lines for OS xenograft models in the included studies. Among studies reported miRNAs as tumor suppressors,data from the 11 studies [36, 38-41, 53, 54, 57-59, 65], which used MG-63 for OS xenograft model, were combined together for the meta-analysis, and there were 72 mice in the intervention arm and 91 mice in the control arms. Tumor volume significantly decreased by rescuing the downregulated miRNAs (pooled MD = [-5.46]; 95% confidence interval [CI]: [-7.03]- [-3.90]; *p* < 0.00001; Figure 11A, part 1) in a random-effects model; data from the 6 studies [40, 41, 44, 45, 52, 60], which used U2 OS for OS xenograft model, were combined together for the meta-analysis, and there were 34 mice in both the intervention and control arms. Tumor volume significantly decreased by rescuing the downregulated miRNAs (pooled MD = [-3.49]; 95% confidence interval [CI]: [-5.36]- [-1.62]; *p* = 0.0004; Figure 11A, part 2) in a random-effects model; data from the 4 studies [36, 55, 56, 63], which used Saos-2 for OS xenograft model, were combined together for the meta-analysis, and there were 23 mice in the intervention arm and 41 mice in the control arm. Tumor volume significantly decreased by rescuing the downregulated miRNAs (pooled MD = [-5.42]; 95% confidence interval [CI]: [-6.63]- [-4.22]; *p* = 0.80; Figure 11A, part 3) in a random-effects model; data from the 3 studies [37, 47, 61], which used 143B for OS xenograft model, were combined together for the meta-analysis, and there were 17 mice in both the intervention and control arms. Tumor volume significantly decreased by rescuing the downregulated miRNAs (pooled MD = [-4.23]; 95% confidence interval [CI]: [-6.05]- [-2.42]; *p* = 0.23; Figure 11A, part 4) in a random-effects model; data from the 3 studies [33, 42, 62], which used SOSP-9607 for OS xenograft model, were combined together for the meta-analysis, and there were 26 mice in the intervention arm and 36 mice in the control arm. Tumor volume significantly decreased by rescuing the downregulated miRNAs (pooled MD = [-4.42]; 95% confidence interval [CI]: [-5.42]- [-3.41]; *p* = 0.91; Figure 11A, part5) in a random-effects model.

As we could see in Figure 11A, the effects on reducing the tumor volume was best when the OS xenograft models were produced by injection of MG-63, then Saos-2, and followed by 143B, U2 OS or SOSP-9607.

There were 2 studies reported tumor volume as the major outcome measure and miRNAs as oncogenes, used Saos-2 for OS xenograft model, were combined together for the meta-analysis, and there were 10 mice in both the intervention and control arms [34, 50]. Tumor volume significantly decreased when the aberrantly expressed miRNAs were corrected (pooled MD = [5.65]; 95% confidence interval [CI]: [3.23]- [8.07]; *p* = 0.72; Figure 11B).

## DISCUSSION

The functional contributions of miRNAs in the development and progression of malignancies have resulted in the development of new therapeutic approaches. Strategies include blocking the up-regulated oncogenic miRNAs using antisense oligonucleotides, or rescuing the downregulated cancer suppressor miRNAs by miRNA mimics [66]. MiRNAs also could be injected into the systemic circulation or introduced into the body (such as into a limb or the peritoneal cavity) or directly injected into a tumor mass [66]. On the other hand, the therapeutic agent to correct the miRNAs being abnormally regulated could be introduced into progenitor or stem cells that would be transplanted subsequently [67].

Several papers have reported that in the *in vivo* models, miRNAs could delay tumor formation and resulted in significantly smaller tumors when transfected into OS cells, compared with non-transfected cells. As well, systemic injection of miRNA/atelocollagen complexes could avoid spontaneous lung metastases in OS [17, 32-45, 63-65]. These results suggest the potential for miRNAs to be used as therapeutic targets for OS.

Since the understanding of the function of miRNAs in OS remains inadequate, we don't know if miRNAs could be directly used for the treatment of patients with OS.

In researches aimed at improving human health care, animal studies still play a crucial role in creating hypotheses that sheds light on the test in preventative or therapeutic clinical trials of new potential interventions. The underlying principle for use of animal studies is to minimize the risks to patients, since only interventions estimated prospectively safe and effective are eventually moved into clinical trials [68, 69].

However, the results usually vary from one study to the next, the conclusions and interpretation are not always straightforward, also no single study is executed flawlessly in all steps, the decisions about effectiveness of an intervention or validity of a hypothesis cannot be based on the results of a single animal study. Therefore, a mechanism is required to pool together the data across studies [68, 70-73].

This is the first meta-analysis to summarize the pre-clinical data and evaluate the potential value of miRNAs as therapeutic targets for OS. We carried out a systematic literature search that included both English and Chinese databases to make sure the comprehensiveness of the studies that were assessed. Two reviewers separately reviewed the studies, evaluated methodological quality, and extracted the data to evade the bias. This is not a comprehensive list of all therapies that has ever been tried in pre-clinical models of OS, but rather, a systematic review and meta-analysis of specific therapies that are being considered for human translation.

However, the reliability of experimental conclusions depends on the high quality experimental design, analysis and reporting. Bias occurs in the results of an animal study or the conclusions drawn from it when a systematic error exists. There are a large number of potential sources of bias, and the risks of selection bias and measurement bias, which are the most important bias, may be diminished through simple study design features, such as randomisation and blinded assessment of outcomes [70, 74]. Unfortunately, previous research has recognized a low popularity of reporting measures to reduce the risk of bias for specific animal disease models [75-80].Failure to depict the research methods and report data properly consequently has potential scientific, ethical, and economic meanings for the entire research procedure and the reputation of those involved in it. This is particularly right for animal research. The ARRIVE (Animal Research: Reporting of *In Vivo* Experiments) guidelines were developed to promote high-quality, comprehensive reporting to allow an precise critical review of what was done and found in the animal researches, which includes a checklist of 20 items describing the minimum information that all scientific publications reporting research using animals should include, for example the number and specific animal characteristics (species, strain, sex, and genetic background); detail information of housing and husbandry; and methods of the experiments, statistics and analyses (including detail methods used to reduce the bias such as randomization and blinding).”

The limitations of the included original studies were also shared in this study as any other meta-analysis. Though we searched both English and Chinese databases, we cannot confirm that all the related studies have been found. Moreover, other important reasons of bias that must be considered are discriminating reporting and publishing [81], since positive data are more likely to be published, the estimations may be overstated due to the evidence for publication bias. Meanwhile, papers published in other languages beyond English and Chinese had not been included in this manuscript due to the language barrier.

We retrieved 36 studies which met the inclusion criteria in this paper, and methodological quality of these included papers was assessed with a component method like that recommended by The Cochrane Collaboration in assessing risk of bias [82]. As we could see in Table 2, all studies reported details on the experimental procedures and animal numbers and strains used were reported except which was not clear in 1 study. Animal ages were reported in most of the studies and animal genders were reported in almost half of the studies. 23 publications described the reporting potential conflicts of interest and study funding. While only six studies reported randomization, one study reported inclusion/exclusion criteria and blinded assessment of outcome; no included studies described allocation concealment, sample-size calculation and reporting of animals excluded from analysis. The absence of above information could be caused by the real flaws in the experimental design or reporting omissions. Therefore, efforts should be made in the future to improve bioscience research design and reporting, such as using the ARRIVE guidelines.

Heterogeneity is acceptable in a meta-analysis, it would be surprising if many studies were completed by different groups in different places with different methods, all of them ended up by measuring the same fundamental parameters. Furthermore, animal studies are usually small (with a sample size of about 10 in each group). Therefore, the challenge is to decide on the most fitting approach to evaluate heterogeneous studies. When heterogeneity cannot be ignored, one analytical technique is to integrate the data into a random-effects model, which involves a hypothesis that the effects being estimated in diverse studies are not equal, however follow some distribution [83, 84]” . And more studies on a single micRNA intervention for OS are necessary in the future.

As the data of our meta-analyses were highly heterogeneous in terms of different cell lines and injection sites of OS cells used for generating animal models, name and function of miRNAs in the pathogenesis of OS, and different vectors were used for microRNA delivery, delaminating analyses were performed based on each of these heterogeneities to allow for evaluation of the distribution of true effects.

By the delaminating analyses based on the factors above, we further explored the effects on reducing the tumor growth, by alteration of the aberrantly expressed miRNAs. Once tumor weight and tumor volume both were taken into account, our results demonstrated that the anti-osteosarcoma effects were the best when miRNAs were infected into OS cells with lentivirus vectors, the up-regulated oncogene miR-214 was corrected or OS xenograft models were produced by subcutaneous injection. However, the inhibitory effect on tumor growth was proved to be the most poor when the OS xenograft models were produced by injection of SOSP-9607 cells. These data indicated that the therapeutic effects, by correcting the aberrantly expressed miRNAs, on OS were closely associated with the route of miRNAs being interfered, which specific miRNA being involved and the original location of the OS, and also the specific pathological type of OS. This implies intervention effect of different miRNA may possess specificity in different pathological or different original OS. So as this work provides a theoretical basis for the future individualized treatment endeavor. Nevertheless, further studies on the inherent correlation between specific miRNA and OS pathological type are necessary.

The results of this meta-analysis suggest that miRNAs are potential therapeutic targets for OS. Our results illustrated a framework for the design of animal studies and clinical trials, and for an evidence-based way to the development of new therapeutics for OS in the future. Moreover, more animal studies with the rigorous design must be carried out, wide-ranging preclinical safety and toxicity studies would be needed before a miRNA-based treatment could be translated from animal studies to human use.

## MATERIALS AND METHODS

### Literature search strategy and selection criteria

We systematically searched 7 databases including PubMed, Web of Science, Embase, Wan Fang Database, China Knowledge Resource Integrated Database, VIP Database, and Chinese BioMedical since their initiation date to May 10, 2016, without restrictions of the languages, publication status or publication dates. The search strategy included the fol­lowing terms: (MicroRNA OR miRNA) AND osteosarcoma AND (mice OR mouse).

Two reviewers (J.L.C. and Y.M.L.) independently selected the literatures by reviewing the titles, abstracts and full texts according to the eligibility criteria. Disagreements were determined by agreement with a third author (Y.P.Y.). Only studies satisfied the criteria were included in the meta-analysis.

### Eligibility criteria

#### Types of studies

Controlled studies that estimate the therapeutic effects on OS in mouse models by correcting the abnormally expressed miRNAs were searched. All studies only having *in vitro* research data and clinical case reporters were excluded.

#### Types of participants

Any gender, any age, or any strain of laboratory mice inoculated with OS cells *via* subcutaneous or intratibial injection.

#### Types of intervention

Any method for correcting the altered miRNA expression of OS in mouse models was included.

### Type of outcome measure

Xenograft models, derived by inoculation of human cancer cells including ectopic xenograft and orthotopic xenograft according to the transplant site, play critical roles in screening new anticancer agents, evaluation the therapeutic efficacy and toxicity. Standard animal models could save money and time, and afford evidence to support clinical trials for anticancer reagents discovery [85]. Tumor volume and tumor weight are indicators used for assessing the anticancer efficacy of anticancer reagents in cancer xenograft models. Xenograft models included in this meta-analysis were produced by subcutaneous or intratibial inoculation of OS cells.

#### Tumor volume

Tumor volume was calculated according to the digital vernier caliper measurements using the following formula: 0.5×a ×b, where a is the largest dimension and b is the square of perpendicular diameter [86].

#### Tumor weight

Tumors were removed and weighed when mice were sacrificed at the end of experiments.

### Data extraction

The details were extracted from the included studies independently by two authors (J.L.C. and Y.M.L.) in this meta-analysis, which included first author name, publication year, information of mice (strain, age and gender), number of mice in each group, method used to produce OS mouse model, how the abnormally expressed miRNA was corrected, and the primary measured outcomes. Data were collected by mean outcome and standard deviation (SD) for each comparison. All data showed by graphs only without numbers were estimated by GetData Graph Digitizer 2.24. A third reviewer (Y.P.Y.) determined any disagreements between the two reviewers.

### Evaluation of methodological quality in the individual study

There are no established dependable and valid tools for the judgment of the methodological quality in animal studies. STAIR (the initial Stroke Therapy Academic Industry Roundtable) was used to evaluate the reporting and design quality of the included studies, which includes: 1) sample-size calculation; 2) inclusion and exclusion criteria; 3) randomization; 4) allocation concealment; 5) reporting of animals excluded from analysis; 6) blinded assessment of outcome; and 7) reporting potential conflicts of interest and study funding [87], and was updated by the STAIR group in 2009 based on the Recommendations for Ensuring Good Scientific Inquiry for America. Two authors (Q.S. and Y.J.W.) assessed the methodological qualities in all included studies and presented as a yes or no. The ‘‘unclear’’ signified that the methodological quality was not clear.

### Statistical analysis

Data were pooled together for analysis if outcomes were reported by 2 or more studies. Two primary outcomes (tumor volume and tumor weight) were analyzed individually. We conducted pair-wise meta-analysis for studies, which directly compared the influence on tumor growth between corrected miRNAs expression and the control (abnormally expressed miRNAs),to determine the pooled relative effect of each intervention compared with the other effect for each measurement outcome of interest, and the mean differences (MDs) of the post-intervention values from the different interventions were determined. We adopted the post-intervention values in meta-analysis derived from the baseline values being comparable between target miRNA group and mimic miRNA or placebo control group, as specified by a Cochrane review [88].

Final consequences from the studies to evaluate differences between the intervention and control group were analyzed by the REVIEW MANAGER 5.1.2 software offered by the Cochrane Collaboration, and I^2 ^was calculated to evaluate the heterogeneity. Heterogeneity was existed if the *p* value was less than 0.10 by the chi-square (x^2^) test. If the I^2^ value was greater than 50%, the results were thought to have a high level of heterogeneity [88]. Clinically and statistically homogeneous studies should be pooled using the fixed-effects model [88]. Clinically homogeneous and statistically heterogeneous studies should be pooled using the random-effects model [88]. When same outcomes were measured using different instruments across studies, we used a standardized mean difference (SMD) in the meta-analysis to combine continuous data.
